# Stepping up local efforts to stop global spread of yellow fever

**DOI:** 10.2471/BLT.18.020618

**Published:** 2018-06-01

## Abstract

A series of outbreaks of yellow fever has triggered the need for a global response. Tatum Anderson reports.

Nigeria has been fighting a major outbreak of yellow fever since September last year, with 358 suspected yellow fever cases and 45 deaths since January. On the other side of the world, Brazil is facing an upsurge of the mosquito-borne disease with unusual patterns of spread and more than 2000 cases since 2016. 

 Yellow fever – which is endemic in many tropical parts of Africa and Latin America – has surged in Brazil’s Amazon rainforest, and, for the first time since the 1940s, surfaced at the doorstep of large urban areas on its Atlantic rim.

Patterns of urban transmission, where the viral haemorrhagic disease quickly amplifies and spreads, were also seen in Africa, during the 2016 outbreaks in Angola, and, its neighbour, the Democratic Republic of the Congo (DRC). 

“We need to think global. The current epidemic risk in Nigeria and Brazil is not only a risk to people in these large countries, but to the whole world,” says Dr Laurence Cibrelus, from the high-threat pathogens unit at the World Health Organization (WHO) in Geneva.

“Recent dengue and Zika epidemics in the Asia–Pacific region indicate the potential risk of the spread of yellow fever to Asian-Pacific countries,” she says, noting that all three viruses are transmitted to humans by the *Aedes aegypti *mosquito.

At a meeting in the Nigerian capital Abuja in April, WHO, the United Nations Children’s Fund (UNICEF) and Gavi, the Vaccine Alliance, re-affirmed their commitment to the* Global strategy to eliminate yellow fever epidemics* (EYE) – adopted in 2016 – with a new initiative to vaccinate 1.4 billion people in 40 countries by 2026. They were joined by officials from 11 African countries, where strategy implementation is a priority. 

Last month, an editorial and research paper in the *Bulletin* highlighted the potential of the yellow fever virus to spread in Asia – where 2 billion people with no immunity to the virus, live in densely populated areas alongside *Aedes aegypti *– with potentially catastrophic consequences.

Successful vaccine campaigns of the 1950s and 1960s in the tropics led to complacency, poor compliance with vaccination requirements in endemic countries and weak enforcement of the International Health Regulations, under which governments must require incoming travellers from endemic countries to provide valid proof of vaccination. 

These and other problems are addressed by the EYE strategy, which combines preventive mass vaccination campaigns and introduction or strengthening of routine immunization, aiming for at least 80% coverage to achieve herd immunity, and strengthening surveillance with laboratory confirmation at regional and national levels.

“Without reliable surveillance and an effective laboratory system, the EYE strategy is blind.”

Oyewale Tomori

Of the 40 countries covered by the strategy, 16 have implemented preventive mass campaigns and 35 include yellow fever in their routine immunization programmes. Routine immunization is a core component of the strategy, because a single dose of the vaccine confers life-long protection. 

“Nigeria has made impressive progress in implementing the strategy,” Cibrelus says, “and, of course, it’s a top priority, because of its large population, including urban populations that have no immunity.”

Nigeria has also drawn up a nine-year plan to extend vaccination coverage to all of its 36 states by 2025, while making a huge commitment to strengthening surveillance and laboratory testing. 

By the end of this year, nine Nigerian states will be covered, says Kazeem Akintunde, a special assistant to Nigeria’s health minister, Professor Isaac Adewole.

Nigeria already includes the yellow fever vaccine in its routine childhood immunization programme, but coverage is uneven and low at 33%, notes Nigerian virologist Oyewale Tomori. 

“We have about two million children every year who have not been vaccinated for the last 10 years,” he says. “This is a huge number of people who are susceptible to the disease.”

 The results of persistently weak yellow fever vaccination coverage are clear. In response to the outbreak last year, Nigeria launched an emergency campaign in which 3 million people were vaccinated. Now a further 8 million people are being vaccinated in a three-round preventive mass campaign in affected states. 

For Tomori, it is not only the weakness of routine yellow fever vaccination that makes Nigeria a priority for the EYE strategy. 

Firstly, following the 2005 Yellow Fever Initiative when preventive mass vaccination was carried out followed by routine immunization in Benin, Gambia, Togo and other west African countries, outbreaks there subsided. At that time, however, only three Nigerian states vaccinated their populations, leaving most Nigerians unprotected.

Then, yellow fever spread eastwards to countries that did not join the 2005 campaign, leading to the 2016 outbreaks in Angola and DRC.

“The fear is that because Nigeria did not complete the preventive mass campaign back then, the next epidemic to spread internationally could start here,” Tomori explains.

Secondly, most childhood vaccinations take place together when babies are small. The yellow fever and measles vaccines are usually given simultaneously later, when a child is 9 months old, but infants are not always brought back and when they are, often the vaccine has run out.

The first phase of Nigeria’s preventive mass campaign reached 10.4 million people in 2014, but was abandoned because of vaccine stockouts at global level. 

And Nigeria faces other challenges. “Our surveillance is weak, so we may have outbreaks but don’t always detect them,” Tomori explains. Moreover, when cases are reported, unreliable laboratory tests lead to misdiagnosis.

“For a country as large as Nigeria, one or two laboratories with capacity for yellow fever diagnosis are not enough. We need laboratory testing decentralized to where the cases occur, to allow for rapid detection and early control measures for outbreak prevention,” Tomori says, adding: “Without reliable surveillance and an effective laboratory system, the EYE strategy is blind.”

Demand for the yellow fever vaccine has soared, and according to Dr Stephen Sosler, Technical Advisor at Gavi, manufacturers have increased production to meet that demand since 2014.

By working with manufacturers and mapping risks to better forecast demand, Sosler is optimistic that the required 1.4 billion yellow fever vaccine doses will be delivered to Nigeria and the other countries covered by the strategy by 2026.

“As long as we don’t have any more massive outbreaks, there’s enough global stock for routine programmes and to continue phased preventive mass campaigns in the most at-risk countries,” Sosler says.

Fifteen of the 34 countries that already include the vaccine in their routine immunization programmes reported stock-outs that affected national coverage between 2013 and 2015.

“Maintaining a reliable global supply will encourage countries that haven’t yet introduced routine programmes to do so,” Sosler says.

Currently only four yellow fever vaccine manufacturers are prequalified by WHO: Brazil’s Bio-Manguinhos, the Russian Federation’s Institute of Poliomyelitis and Viral Encephalitides, Senegal’s Institut Pasteur and France’s Sanofi Pasteur.

“What we need now is the political will to commit the necessary resources to reach these communities.”

Yodit Sahlemariam

Countries that are eligible for Gavi funding, such as Nigeria, cover the cost of vaccine for routine immunization themselves, while vaccine for mass preventive campaigns is funded by Gavi. Middle-income countries covered by the EYE strategy that are not Gavi eligible, can procure the vaccine at a low price negotiated by UNICEF. 

In addition, emergency vaccine supplies for outbreaks are provided by the Gavi-funded International Coordination Group (ICG) on request to any country in need.

According to Sosler, only 3 of the 4 prequalified manufacturers are supplying the ICG stockpile, because Bio-Manguinhos has prioritized its supply to respond to the outbreak in Brazil. The IGC, which is automatically replenished, has nevertheless maintained its required stock of six million doses for emergencies. 

In Brazil, 21.6 million people have been vaccinated in preventive mass campaigns in São Paulo, Rio de Janeiro and Bahia states this year, according to Carla Domingues, head of the health ministry’s General Coordination of the National Immunization Programme.

Mop-up campaigns in 3 further states, Santa Catarina, Parana and Rio Grande do Sud, should be completed by the end of this year. Next year the campaign will target Brazil’s northeast, she says.

In addition, routine childhood yellow fever vaccination, which has been recommended in 3560 municipalities, is being extended to 2200 municipalities to cover the whole country.

“By the end of 2019, the vaccine will have been offered to the entire Brazilian population,” Domingues says.

In February, a preventive mass campaign was held in subway stations across São Paulo state using fractional doses. “This strategy was used to vaccinate the largest number of people living in large centres in a short time,” she says. 

Studies show that one fifth of the regular yellow fever vaccine dose provides full immunity for at least 12 months. Such dose sparing is used in outbreaks when vaccine supplies are limited.

Like Nigeria, Brazil’s efforts are also fraught with challenges, particularly producing adequate vaccine supplies. Later this year Brazil is opening a new plant to boost production.

“The second challenge is people’s fear of vaccination,” Domingues says, explaining that adults are used taking their children for vaccination but not receiving jabs themselves. The risk of death from an adverse vaccine reaction is about 1 in a range of 0.5 million to 3 million, Domingues says, adding that the authorities are working hard to explain that this risk is so much less than the risk of death from yellow fever itself. In Brazil’s current outbreak nearly 40% of cases have been fatal, she says.

UNICEF is leading advocacy and communications efforts for the roll-out of the EYE strategy at the global and country levels. “The technical solutions for achieving high population immunity among the at-risk populations are known,” says Dr Yodit Sahlemariam from UNICEF. “What we need now is the political will to commit the necessary resources to reach these communities,” she says.

**Figure:**
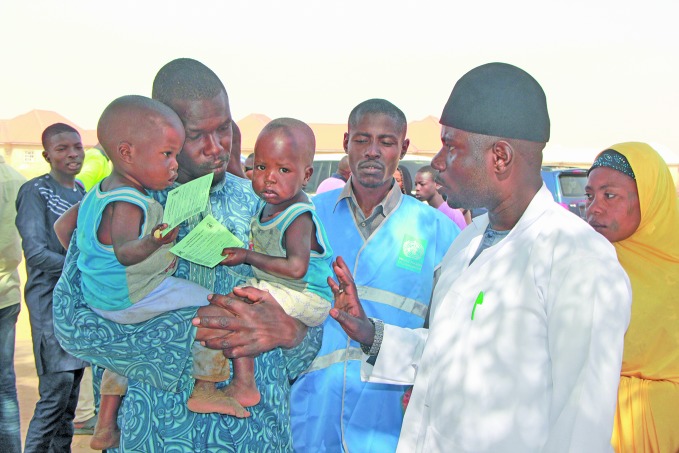
Yellow fever vaccination campaign in Borno state, Nigeria, in February 2018.

WHO/CE Onuekwe

